# Disease spectrum of myopathies with elevated aldolase and normal creatine kinase

**DOI:** 10.1111/ene.16117

**Published:** 2023-11-03

**Authors:** Pannathat Soontrapa, Shelly Shahar, Lattawat Eauchai, Floranne C. Ernste, Teerin Liewluck

**Affiliations:** ^1^ Department of Neurology, Division of Neuromuscular Medicine Mayo Clinic Rochester Minnesota USA; ^2^ Department of Medicine, Division of Neurology, Siriraj Hospital Mahidol University Bangkok Thailand; ^3^ Department of Neurology Rambam Health Care Campus Haifa Israel; ^4^ Bruce Rappaport Faculty of Medicine Technion‐Israel Institute of Technology Haifa Israel; ^5^ Department of Anatomy, Siriraj Hospital Mahidol University Bangkok Thailand; ^6^ Department of Medicine, Division of Rheumatology Mayo Clinic Rochester Minnesota USA

**Keywords:** dermatomyositis, elevated aldolase, myopathies, normal creatine kinase, perimysial pathology

## Abstract

**Background and purpose:**

Elevation of serum creatine kinase (CK) or hyperCKemia is considered a biological marker of myopathies. However, selective elevation of serum aldolase with normal CK has been reported in a few myopathies, including dermatomyositis, immune‐mediated myopathy with perimysial pathology and fasciitis with associated myopathy. The aim was to investigate the disease spectrum of myopathies with isolated aldolase elevation.

**Methods:**

Medical records were reviewed to identify patients >18 years old seen between December 1994 and June 2020 who had pathologically proven myopathies with elevated aldolase and normal CK level. Patients with alternative causes of aldolase elevation were excluded.

**Results:**

Thirty‐four patients with various types of myopathies were identified. Myopathies were treatable in 27 patients. The three most common etiologies were dermatomyositis (*n* = 8), overlap myositis (*n* = 4) and nonspecific myopathy (*n* = 4). Perimysial pathology comprising inflammation, fragmentation, vasculitis, calcified perimysial vessels or extracellular amyloid deposition was found in 17/34 patients (50%). Eight dermatomyositis patients with selective elevated aldolase were compared to 24 sex‐ and age‐matched patients with dermatomyositis and hyperCKemia. Dermatomyositis patients with normal CK significantly (*p* < 0.05) had less frequent cutaneous involvement (50.0% vs. 100.0%) and fibrillation potentials (50.0% vs. 90.5%) but higher median erythrocyte sedimentation rate (33.5 vs. 13.5 mm/h) and more common perifascicular mitochondrial pathology (37.5% vs. 4.2%).

**Conclusion:**

Isolated aldolase elevation can be found in a greater variety of myopathies than initially thought and most were treatable. Dermatomyositis is the most common myopathy with selective elevation of aldolase in our cohort, which features some unique characteristics compared to dermatomyositis with hyperCKemia.

## INTRODUCTION

Creatine kinase (CK) is a muscle enzyme involved in the phosphocreatine circuit for energy supply of muscles [1], whilst aldolase is a glycolytic enzyme abundantly expressed in not only muscles but also liver, brain and red blood cells [2]. Elevation of serum CK (hyperCKemia) and aldolase level is considered a biomarker of muscle damage secondary to a leakage of these cytosolic enzymes into the bloodstream [3]. HyperCKemia is often accompanied by high aldolase level [4, 5]. The magnitude of CK elevation generally directly correlates with the number of necrotic fibers [6, 7]. CK level can be normal in myopathies without prominent necrotizing pathology, for example congenital myopathies or metabolic myopathies [8, 9]. Immune‐mediated myopathies generally feature a significant degree of necrotic fibers and CK elevation; however, a proportion of patients with dermatomyositis, inflammatory myopathy or polymyositis, inflammatory myopathy with perimysial pathology, scleroderma myopathy and eosinophilic fasciitis can have normal serum CK levels with elevated aldolase levels [10, 11, 12, 13, 14, 15, 16, 17]. In skeletal muscle, aldolase is abundantly expressed in myoblasts in the early stage of differentiation and in regenerating fibers, whilst CK expression in these cells is minimal to none. It is hypothesized that immune‐mediated destruction of these regenerating myofibers may underlie the discrepancy of aldolase elevation and normal CK level [18]. In some patients in whom myodegeneration is not a prominent histopathological feature (e.g., eosinophilic fasciitis), aldolase may be derived from cells other than myofibers [14, 19, 20]. Herein, the aim was to investigate the disease spectrum of myopathies with elevated aldolase and normal CK level.

## METHODS

The Mayo Clinic database was reviewed to identify patients aged 18 or older seen between 1 December 1994 and 30 June 2020, who were diagnosed with myopathy based on electromyography and had elevated aldolase with normal CK at the time of muscle biopsy. The normal CK range is 39–308 U/L in men and 26–192 U/L in women. Elevated aldolase is defined as an aldolase level ≥7.7 U/L. Only patients who had myopathological evidence of myopathy were included. Patients were excluded if they had one or more of the following features: concomitant liver disease based on liver biopsy, hepatic ultrasound or elevated gamma‐glutamyl transferase, hemolytic anemia, or insufficient clinical information. Patients were diagnosed with nonspecific myositis when muscle biopsy showed significant inflammatory changes without characteristic features of dermatomyositis or inclusion body myositis but overlap myositis or antisynthetase syndrome cannot be entirely excluded due to inadequate clinical or serological information. Nonspecific myopathy was defined as muscle biopsy showing nonspecific myopathic features, for example increased internal nuclei, fiber splitting, and rare necrotic or regenerating fibers without inflammatory changes. The number of necrotic fibers in these patients was too infrequent to be categorized as an active myopathy or necrotizing myopathy. The absence of inflammatory changes helped distinguish them from nonspecifc myositis. Clinical information and results of ancillary tests in each patient were reviewed. Clinical improvement was defined as at least one level of improvement on the modified Rankin scale. The study was approved by the institutional review board of Mayo Clinic.

### Statistical analysis

For analytic data, chi‐squared or Fisher's exact tests were used to compare between two discrete variables. The Wilcoxon rank sum test was used to compare between two continuous variables. A *p* value <0.05 was considered statistically significant.

## RESULTS

**FIGURE 1:**
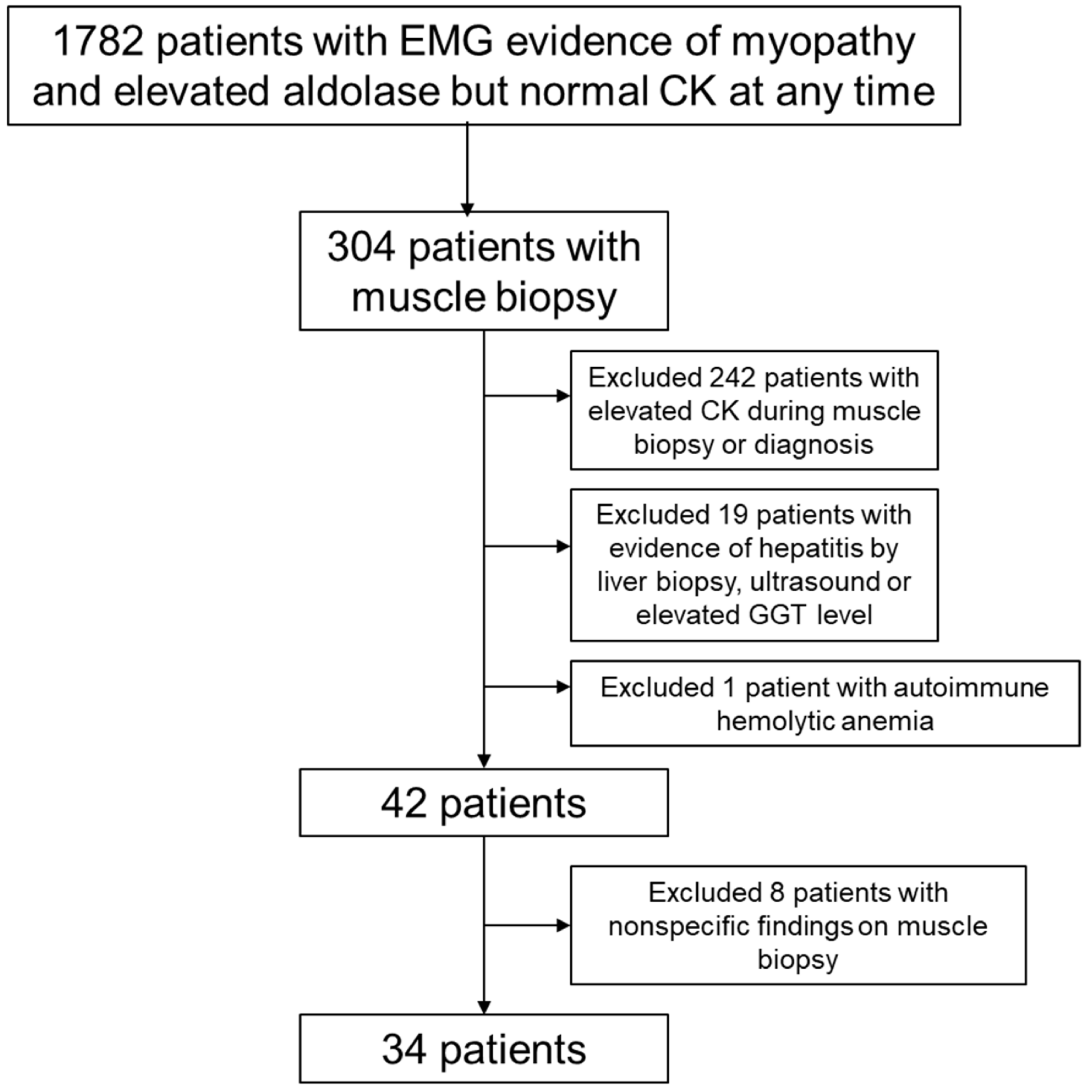
A flowchart of patient inclusion.

**FIGURE 2:**
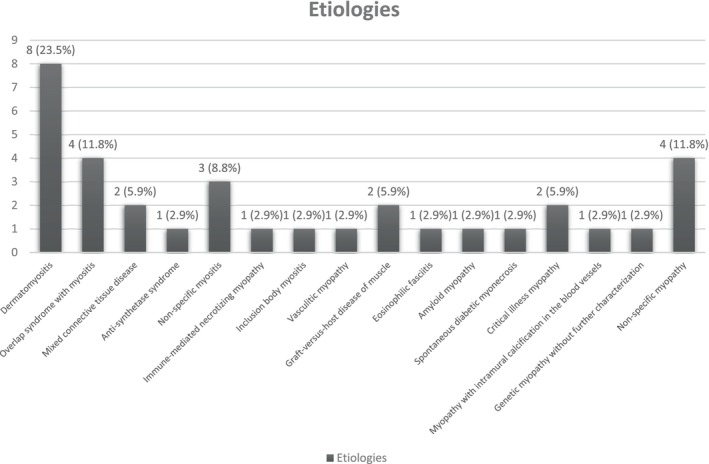
Diagnosis of myopathy patients with isolated elevation of serum aldolase.

There were 1782 patients with electromyography evidence of myopathy and elevated aldolase with normal CK. Myopathic changes were observed on muscle biopsy in 296 patients from a total of 304 patients who underwent muscle biopsy. A total of 262 patients (242 patients with elevated CK at the time of muscle biopsy, 19 patients with concomitant liver diseases and one patient with autoimmune hemolytic anemia) were excluded (Figure 1). Thirty‐four patients were included (Figure 2; Table 1). Median aldolase level was 10.0 U/L (interquartile range 8.8, 11.8). Median CK level was 67.0 U/L (interquartile range 33.0, 116.5). The three most common etiologies were dermatomyositis (*n* = 8), overlap myositis (*n* = 4, including two systemic lupus erythematosus, one rheumatoid arthritis and one Sjögren's syndrome and sarcoidosis) and nonspecific myopathy (*n* = 4). Four patients were diagnosed with nonspecific myopathy, three of whom received prednisone 5–34 months prior to the muscle biopsy. Of note, a patient with immune mediated necrotizing myopathy had weakness and hyperCKemia 7.5 years before the diagnosis and was found to have end‐stage muscle on biopsy and elevated aldolase in isolation at the time of biopsy.

**TABLE 1:** Demographic data, clinical presentations, laboratory findings and clinical course of 34 patients with myopathy and isolated aldolase elevation.

Features	Number of patients (%)
Sex, male	10 (29.4%)
Age at diagnosis, years, median (IQR)	63.9 (50.1, 68.9)
Age at symptom onset, years, median (IQR)	60.6 (45.7, 69.0)
Underlying autoimmune diseases^a^	13 (38.3%)
Muscle symptoms	
Myalgia	13 (38.3%)
Proximal muscle weakness	34 (100.0%)
Distal muscle weakness^b^	8 (23.5%)
Muscle cramps	1 (2.9%)
Muscle atrophy	5 (14.7%)
Muscle biopsy findings	
Perimysial pathology^c^	17 (50.0%)
Perifascicular pathology^d^	8 (23.5%)
Perifascicular atrophy	7 (20.6%)
Presence of necrotic fibers	26 (76.5%)
Perifascicular necrotic fibers	6 (17.6%)
Necrotic fibers deeper in fascicles	25 (73.5%)
Presence of regenerating fibers	26 (76.5%)
Perifascicular regenerating fibers	6 (17.6%)
Regenerating fibers deeper in fascicles	23 (67.6%)
Perifascicular COX negative fibers	3 (8.8%)
Perifascicular vacuolar changes	4 (11.8%)
Treatment	
Immunomodulatory therapy^e^	24/31 (77.4%)
Clinical improvement at 6 months^f^	16/24 (66.7%)

Abbreviations: COX, cytochrome c oxidase; IQR, interquartile range.

^a^ Underlying autoimmune diseases include five rheumatoid arthritis, four Sjögren's syndrome, three systemic lupus erythematosus, two mixed connective tissue disease, one undifferentiated connective tissue disease, one sarcoidosis and one scleroderma.

^b^ Distal muscle weakness was found in one inclusion body myositis, one immune‐mediated necrotizing myopathy, one critical illness myopathy, two nonspecific myopathy, one amyloid myopathy, one overlap myositis and one myopathy with intramural calcification of the blood vessels.

^c^ Perimysial pathology was found in seven dermatomyositis, one perifascicular pathology without inflammation, three inflammatory myopathy with perimysial inflammation, one vasculitic myopathy, one mixed connective tissue disease, one graft‐versus‐host disease, one light chain amyloid myopathy, one myopathy with intramural calcification of perimysial vessels and one eosinophilic fasciitis.

^d^ Perifascicular pathology including any of perifascicular necrosis, regeneration and atrophy was observed in eight patients (six dermatomyositis, one overlap myositis and one myopathy with intramural calcification of the blood vessels).

^e^ Unknown treatment in three patients.

^f^ No follow‐up data in eight patients and two patients died.

All 34 patients had predominant proximal muscle weakness, eight of whom also had mild distal weakness. Eight patients were wheelchair dependent or had modified Rankin scale >3. Muscle biopsy showed perimysial pathology in 17 patients, seven of whom had non‐dermatomyositis and non‐inflammatory myopathy with perimysial pathology diagnosis (one mixed connective tissue disease, one graft‐versus‐host disease, one vasculitic myopathy, one light chain amyloid myopathy, one myopathy with intramural calcification of the blood vessels, one eosinophilic fasciitis with rare necrotic fibers, and one perifascicular pathology without inflammation). Perifascicular pathology was observed in eight patients. Twenty‐four patients received immunomodulatory therapy, which led to improvement in 16 patients at 5–7 months from the diagnosis. Nine of 17 patients (52%) had normalization of aldolase by 6 months (Table S1).

The included dermatomyositis patients were further compared to 24 sex‐ and age‐ matched dermatomyositis patients with elevated CK (Table 2). Elevated aldolase was found in all 19 tested dermatomyositis patients with hyperCKemia. Dermatomyositis patients with hyperCKemia significantly (*p* < 0.05) had more frequent cutaneous findings and fibrillation potentials and lower median erythrocyte sedimentation rate (ESR) than dermatomyositis patients with normal CK. Perifascicular cytochrome c oxidase (COX) negative fibers were significantly more common in the normal CK group than the other (*p* = 0.04). There were no significant differences in other perifascicular pathology or treatment responses between dermatomyositis with and without hyperCKemia.

**TABLE 2:** Comparison of sex‐ and age‐matched dermatomyositis patients with and without elevated CK.

Features	Normal CK *N* = 8 (%)	High CK *N* = 24 (%)	*p* value
*Demographic data and clinical presentations*			
Sex, male	3 (37.5%)	9 (37.5%)	1.0
Age at diagnosis, years, median (IQR)	64.4 (51.2, 68.6)	63.6 (50.5, 65.6)	0.79
Underlying autoimmune diseases	3 (37.5%)	2 (8.3%)	0.08
Concurrent malignancy^a^	2 (25.0%)	4 (16.7%)	0.62
Age of symptom onset, median (IQR)	63.4 (48.5, 66.6)	63.3 (49.8, 65.5)	1.00
Time to diagnosis, months, median (IQR)	3.5 (2.0, 5.4)	2.3 (1.4, 4.3)	0.30
Muscle signs or symptoms			
Myalgia	3 (37.5%)	13 (54.2%)	0.41
Proximal muscle weakness	8 (100.0%)	24 (100%)	NA
Distal muscle weakness	0 (0%)	9 (37.5%)	0.07
Facial weakness	1 (12.5%)	2 (8.3%)	0.73
Neck weakness	4 (50.0%)	15 (62.5%)	0.53
Truncal weakness	0 (0%)	3 (12.5%)	0.55
Truncal or distal weakness	0 (0%)	10 (41.7%)	0.03
Muscle atrophy	0 (0%)	3 (12.5%)	0.55
Muscle stiffness	0 (0%)	1 (4.2%)	1.00
Dysphagia	3 (37.5%)	14 (58.3%)	0.42
Dyspnea	1 (12.5%)	10 (41.7%)	0.13
Interstitial lung disease	0 (0%)^b^	4 (16.7%)	0.22
Concurrent neuropathy	1 (12.5%)	1 (12.5%)	0.44
Associated symptoms	5 (62.5%)	24 (100%)	0.002
Fever	2 (25.0%)	2 (6.2%)	0.25
Constitutional symptoms	3 (37.5%)	8 (33.3%)	0.83
Skin rash	4 (50.0%)	24 (100.0%)	0.002
Pathognomonic skin rash^c^	0 (0%)	8 (33.3%)	0.06
Nonspecific rash	4 (50.0%)	16 (66.7%)	0.43
mRS			
Grade 1	4 (50.0%)	9 (37.5%)	0.68
Grade 2	2 (25.0%)	14 (58.3%)	0.22
Grade 3	2 (25.0%)	1 (4.2%)	0.15
*Laboratory findings*			
Elevated ESR	4/8 (50.0%)	6/22 (27.3%)	0.24
ESR, mm/h, median (IQR)	33.5 (11.5, 69.2)	13.5 (10.0, 22.2)	0.01
Elevated CRP	1/7 (14.3%)	1/18 (5.6%)	0.47
CRP, mg/L, median (IQR)	3.0 (0.1,6.0)	1.3 (0.5,3.8)	0.11
CK, U/L, median (IQR)	64.0 (26.0, 109.8)	1385.5 (1011.8, 3121.2)	0.02
Aldolase, U/L, median (IQR)	10.8 (9.3, 14.2)	19.9 (14.4, 36.5)	0.07
Elevated AST	3/8 (37.5%)	19/23 (82.6%)	0.02
Elevated ALT	4/7 (57.1%)	15/19 (78.9%)	0.27
Myositis specific antibody	2/5 (40.0%)	3/4 (75.5%)	0.29
Anti‐TIF‐1‐gamma	0	0	
Anti‐NXP‐2	1	1	
Anti‐MDA‐5	1	0	
Anti‐Mi2	0	2	
*EMG findings* ^d^			
Myopathic motor unit potentials			
Proximal muscles only	5 (62.5%)	10 (47.6%)	0.47
Distal muscles only	0 (0%)	1 (4.7%)	1.0
Proximal and distal muscles	3 (37.5%)	10 (47.6%)	0.70
Truncal muscles	6 (75.0%)	9 (42.9%)	0.21
Fibrillations ≥1+	4 (50.0%)	19 (90.5%)	0.02
*Muscle pathology*			
Perifascicular pathology^e^	6 (75.0%)	13 (54.2%)	0.42
Perifascicular atrophy	6 (75.0%)	10 (41.7%)	0.22
Perifascicular necrotic fibers	5 (62.5%)	9 (37.5%)	0.25
Perifascicular regenerating fibers	5 (62.5%)	8 (33.3%)	0.22
Perifascicular internalized nuclei	5 (62.5%)	10 (41.7%)	0.42
Perifascicular COX negative fibers	3 (37.5%)	1 (4.2%)	0.04
Perifascicular vacuolar changes	4 (50.0%)	9 (37.5%)	0.68
Perimysial inflammation	5 (62.5%)	9 (37.5%)	0.25
Endomysial inflammation	5 (62.5%)	10 (41.7%)	0.42
Vasculitis	1 (12.5%)	1 (4.2%)	0.44
Increased endomysial connective tissue	4 (50.0%)	4 (16.7%)	0.06
Reduced capillary density	1/1 (100.0%)	4/4 (100.0%)	0.18
Membrane attack complex‐positive capillaries	1/2 (50.0%)	3/5 (60.0%)	0.81
Treatment response			
Improved	6/7 (85.7%)	21/21 (100%)	0.21
Death	1 (12.5%)	8 (33.3%)	0.26
Cancer‐related death	1 (100.0%)	4 (50.0%)	0.34

Abbreviations: ALT, alanine transferase; anti‐MDA‐5, anti‐melanoma differentiation‐associated gene 5; anti‐NXP‐2, anti‐nuclear matrix protein 2; anti‐TIF‐1‐gamma, anti‐transcription intermediary factor 1; AST, aspartate transferase; CK, creatine kinase; COX, cytochrome c oxidase; CRP, C‐reactive protein; CT, computed tomography; EMG, electromyography; ESR, erythrocyte sedimentation rate; IQR, interquartile range; mRS, modified Rankin Scale; NA, not available.

^a^ Diagnosis of malignancy 1 year before or after the diagnosis of dermatomyositis.

^b^ All patients underwent conventional chest CT scan.

^c^ Pathognomonic skin rashes are heliotrope and Gottron papules.

^d^ EMG was not done in three cases of the high CK group.

^e^ Perifascicular pathology includes any of perifascicular necrosis, regeneration and atrophy.

## DISCUSSION

Our findings expand the spectrum of myopathies with elevated aldolase and normal CK levels to include not only dermatomyositis and other inflammatory myopathies but also a variety of other acquired myopathies, as shown in Figure 1, most of which are treatable diseases. This emphasizes the importance of measuring aldolase levels in patients with suspected myopathy, which helps increase the sensitivity of myopathy detection, especially in patients with normal CK levels. It is also important to note that aldolase may remain elevated even in the advanced stage of immune‐mediated necrotizing myopathy or in steroid‐treated patients who had normal CK levels.

Nozaki and Pestronk identified perimysial pathology in 11/12 (92%) patients with myopathy featuring selective elevation of aldolase [16]. In our cohort, combining all conditions that affect the perimysium including any perimysial inflammation, amyloid deposition and vasculitis, such pathology was observed in only 17/34 (50%) patients. Fibrillation potentials were observed in 80% of our patients compared to 18% of patients reported by Nozaki and Pestronk. These discrepancies of myopathology and electrodiagnostic findings could be due to a broader spectrum of myopathies included in our cohort.

In dermatomyositis, CK can be normal in about 10%–26% of patients [17, 21, 22]. Comparing dermatomyositis patients with and without hyperCKemia, few significant differences were found between these two groups. In our cohort, cutaneous involvement was more common in the hyperCKemia group (100% vs. 50%), which is opposite to what was observed in the prior study that all dermatomyositis patients with normal CK (9/9) had rashes [17]. This difference could stem from the different diagnostic criteria for dermatomyositis applied in the study. A pathological diagnosis was used, whilst the previous study used Bohan and Peter criteria [23]. In the Bohan and Peter criteria, rashes are a must for a definitive diagnosis of dermatomyositis if no muscle pathology is available, whilst in fact dermatomyositis patients may have myositis without cutaneous manifestations [24].

Dermatomyositis patients with and without hyperCKemia had proximal weakness. However, only the hyperCKemia group also had mild weakness affecting distal or truncal musculature (*p* = 0.03). Interestingly, the elevated CK group had more frequent fibrillation potentials, which are known to correlate with the presence of necrotic fibers, fiber splitting or vacuolization of muscle fibers [25]. Therefore, the CK elevation in dermatomyositis patients may reflect a more extensive muscle damage and more widespread muscle involvement in dermatomyositis patients with hyperCKemia than in those without. It is unclear why the normal CK group had higher median ESR than the elevated CK group. Interestingly, the elevated ESR in inflammatory myopathies was previously shown to be associated with interstitial lung disease rather than muscle inflammation [26]. However, all eight dermatomyositis patients with normal CK underwent a conventional computed tomography scan of the chest without radiological evidence of interstitial lung disease. On muscle histopathology, the normal CK group had significantly higher numbers of patients with perifascicular COX negative fibers (37.5% vs. 4.2%). A previous study showed that perifascicular mitochondrial pathology occurred in 46.3% of dermatomyositis patients, but its correlation with CK level is unknown [27]. This mitochondrial dysfunction is thought to be a result of interferon‐beta induced reactive oxygen species leading to mitochondrial DNA depletion [28, 29]. Our observation of frequent perifascicular mitochondrial alterations in dermatomyositis patients with normal CK and elevated aldolase requires additional studies in a larger cohort of patients.

The current study has some limitations. First, the sample size was relatively small, although this was the largest cohort of myopathies with selective elevation of aldolase ever reported. Secondly, only a small number of dermatomyositis patients underwent myositis‐specific antibody testing. A larger prospective study with universal testing for myositis‐specific antibodies is crucial to address these issues. Thirdly, only conventional and not high‐resolution chest computed tomography scan was performed in dermatomyositis patients with normal CK, which may lead to the underdiagnosis of minimally symptomatic or asymptomatic interstitial lung disease amongst these patients, especially those with elevated ESR.

In conclusion, an isolated elevation of aldolase can be seen in a broader range of myopathies than was initially thought. Several of these were treatable acquired myopathies. Testing for both aldolase and CK probably increases myopathy detection compared to testing for CK alone. Elevation of CK in dermatomyositis occurs with more frequent cutaneous involvement and fibrillation potentials, as well as more diffuse muscle involvement. A high frequency of perifascicular mitochondrial pathology in dermatomyositis patients without CK elevation is of particular interest and requires a large multicenter study to decipher the underlying mechanism of this observation.

## AUTHOR CONTRIBUTIONS

**Pannathat Soontrapa:** Conceptualization; methodology; investigation; writing – original draft; formal analysis; writing – review and editing; validation. **Lattawat Eauchai:** Investigation. **Floranne C. Ernste:** Writing – review and editing. **Teerin Liewluck:** Conceptualization; methodology; supervision; writing – review and editing; data curation.

## CONFLICT OF INTEREST STATEMENT

It is confirmed that the journal's position on issues involved in ethical publication have been read and this report is consistent with those guidelines. None of the authors has any conflict of interest to disclose.

## Supporting information


Table S1:

## Data Availability

The data that support the findings of this study are available from the corresponding author upon reasonable request.
