# Supplementary material for: Disease spectrum of myopathies with elevated aldolase and normal creatine kinase
Source: Eur J Neurol. 2023 Nov 3;31(2):e16117. doi: 10.1111/ene.16117 (PMC11235866; doi:10.1111/ene.16117)
Supplement: Supplementary file 1 — Table S1: [file ENE-31-e16117-s001.docx]

**Supplementary table 1**: Additional demographic data, clinical presentations, laboratory findings, muscle biopsy and clinical course of cases with isolated serum aldolase elevation.

|  | N= 34 (%) |
| --- | --- |
| **Demographic data and clinical presentations** | |
| Race   - Caucasian - American Indian or Alaska Native - Asian - Hispanic/ Latino - Unknown | 27 (79.4%)  1 (2.9%)  2 (5.9%)  1 (2.9%)  3 (8.8%) |
| Underlying diseases   - Diabetes mellitus - Hyperlipidemia - Hypertension - Chronic kidney disease (GFR < 60 mL/min/1.73m^2^) - Post-transplantation - Pulmonary hypertension - hypothyroidism | 5 (14.7%)  4 (11.8%)  15 (44.1%)  4 (11.8%)  2 (5.9%)  5 (14.7%)  8 (23.5%) |
| Malignancy^a^ | 3 (8.8%) |
| Multiple myeloma | 1 (2.9%) |
| Time to diagnosis, months, median (IQR) | 4.4 (1.4,16.6) |
| Concurrent neuropathy | 14 (41.2%) |
| Other associated symptoms   - Fever - Night sweat - Weight loss - Arthralgia - Arthritis - Raynaud’s phenomenon | 18 (52.9%)  5 (14.7%)  3 (8.8%)  7 (20.6%)  3 (8.8%)  3 (8.8%)  4 (11.8) |
| Skin lesions   - Dermatomyositis hallmark lesions - Rash - Telangiectasia | 13 (38.2%)  0 (0%)  13 (38.2%)  1 (2.9%) |
| Pulmonary involvement   - Interstitial lung - Nonspecific interstitial pneumonia - Diaphragmatic weakness | 7 (20.6%)  3 (8.8%)  3 (8.8%)  1 (2.9%) |
| Cardiomyopathy | 1 (2.9%) |
| Ambulation   - Self-ambulation - Cane - Walker - Wheelchair | 15 (44.1%)  5 (14.7%)  6 (17.6%)  8 (23.5%) |
| mRS   - Grade 1 - Grade 2 - Grade 3 - Grade 4 - Grade 5 | 9 (26.5%)  11 (32.4%)  6 (17.6%)  7 (20.6%)  1 (2.9%) |
| **Laboratory findings** | |
| CK, U/L, median (IQR) | 67.0 (33.0,116.5) |
| Aldolase, U/L, median (IQR) | 10.0 (8.8,11.8) |
| Elevated ESR | 12 (37.5%) |
| ESR, mm/h, median (IQR) | 17.0 (10.0,55.5) |
| Elevated CRP | 9 (31%) |
| CRP, mg/L, median (IQR) | 3.0 (0.3,13.6) |
| Aldolase in each etiology, U/L, median (IQR)   - Dermatomyositis - Overlap syndrome with myositis - Mixed connective tissue disease - Anti-synthetase syndrome - Non-specific myositis - Immune-mediated necrotizing myopathy - Inclusion body myositis - Vasculitic myopathy - Graft-versus-host disease - Eosinophilic fasciitis - Amyloid myopathy - Spontaneous diabetic myonecrosis - Critical illness myopathy - Myopathy with intramural calcification in the blood vessels - Genetically uncharacterized myopathy - Nonspecific myopathy | 10.7 (9.3,14.2)  9.5 (8.7,10.3)  8.2 (8.2,8.3)  8.3 (NA)  13.2 (12.4,16.0)  8.4 (NA)  12.4 (12.4,12.4)  8.2 (NA)  10.3 (9.6,11.0)  9.5 (NA)  11.1 (NA)  9.8 (NA)  12.4 (11.5,13.4)  14.0 (NA)  15.6 (NA)  9.4 (9.1,9.8) |
| Positive ANA | 13 (41.9%) |
| Elevated AST | 12 (35.3%) |
| Elevated ALT | 9 (33.3%) |
| Elevated ALP | 8 (27.6%) |
| Elevated GGT^b^ | 0 (0%) |
| Eosinophilia | 8 (24.2%) |
| Myositis antibodies (tested 17 cases)   - Anti-TIF1 gamma - Anti-NXP-2 - Anti-MDA-5 - Anti-Jo1 - Anti-PM/Scl | 0 (0%)  1 (5.9%)  1 (5.9%)  1 (5.9%)  2 (11.8%) |
| Necrotizing autoimmune antibody (tested 10 cases)   - Anti-HMGCR - Anti-SRP | 1 (10.0%)  0 (0%) |
| Extractable nuclear antigen antibody (tested 30 cases)   - Anti-SSA - Anti-SSB | 7 (23.3%)  3 (10.0%) |
| ANCA (tested 30 cases)   - P-ANCA - C-ANCA | 1 (3.3%)  0 (0%) |
| **EMG findings** | |
| Myopathic units   - Proximal muscles - Distal muscles - Proximal and distal muscles | 19 (55.9%)  1 (2.9%)  14 (41.2%) |
| Truncal involvement | 19 (55.9%) |
| Fibrillations | 27 (79.4%) |
| Fibrillations ≥ 1+^c^ | 24 (70.6%) |
| Mixed small and large units | 7 (20.6%) |
| Presence of concurrent neuropathy   - Axonal sensorimotor neuropathy - Asymmetric polyneuropathy | 14 (41.2%)  1 (2.9%) |
| **Muscle pathology** | |
| Inflammatory collections   - Individual - Small - Moderate - Large - Unspecified | 22 (59.5%)  2 (5.4%)  6 (16.2%)  1 (2.7%)  5 (13.5%)  8 (21.6%) |
| Inflammatory cells invading non-necrotic fibers | 3 (8.8%) |
| Endomysial inflammation | 13 (38.2%) |
| Rimmed vacuoles | 2 (5.9%) |
| Vacuoles in regenerating fibers | 3 (9.1%) |
| Vasculitis^d^ | 4 (11.8%) |
| Vessel hyalinization | 5 (14.7%) |
| Increase in endomysial connective tissue | 11 (32.4%) |
| Mitochondrial dysfunction | 6 (17.6%) |
| Type 1 fiber atrophy | 1 (2.9%) |
| Type 2 fiber atrophy | 14 (41.2%) |
| Denervation atrophy | 18 (52.9%) |
| Reinnervation | 8 (23.5%) |
| Glycogen accumulation | 0 (0%) |
| Congophilic deposits | 1 (2.9%) |
| Intracellular congophilic deposits^e^ | 2 (5.9%) |
| Reduced capillary | 2 (5.9%) |
| MAC staining of capillary | 2 (5.9%) |
| Nerve vasculitis on nerve biopsy (from 3 biopsies) | 1 (33.3%) |
| **Treatment** | |
| Immunotherapy^f^   - Steroid - IVIG - Methotrexate - Azathioprine - Mycophenolate mofetil - Rituximab - Cyclophosphamide (oral) - Infliximab - Cyclosporine - Belimumab - Sirolimus | 25 (73.5%)  25 (73.5%)  5 (14.7%)  8 (23.5%)  7 (20.6%)  9 (26.5%)  4 (11.8%)  1 (2.9%)  1 (2.9%)  1 (2.9%)  1 (2.9%)  1 (2.9%) |
| Ambulation at 6 months after treatment^g^   - Self-ambulation - Cane - Walker - Wheelchair | 13/24 (54.2%)  4/24 (16.7%)  5/24 (20.8%)  2/24 (8.3%) |
| mRS at 6 months^g^   - Grade 0 - Grade 1 - Grade 2 - Grade 3 - Grade 4 | 7/24 (29.2%)  6/24 (25.0%)  4/24 (16.7%)  5/24 (20.8%)  2/24 (8.3%) |
| Normalization of aldolase at 6 months | 9/17 (52.9%) |
| Death  Age of death, median (IQR) | 10 (29.4%)  69.7 (55.3,74.1) |

a: 1 multiple myeloma, 1 papillary thyroid carcinoma, and 1 chondrosarcoma. b: tested in one case. c: fibrillations ≥ 1 means a presence of at least sustained fibrillations in 2 more areas d: 2 graft-versus-host disease, 1 vasculitic myopathy, 1 dermatomyositis. e: 1 in inclusion body myositis and another in 2 fibers. f: no follow-up data in 3 cases. g: no follow-up data in 8 patients and 2 patients expired.

ALP, alkaline phosphatase; ALT, alanine transferase; ANA, antinuclear antibodies; ANCA, antineutrophil cytoplasmic antibodies; AST, aspartate transferase; anti-HMGCR, anti- 3-hydroxy-3-methylglutaryl-CoA reductase; anti-MDA-5, anti-melanoma differentiation-associated gene 5; anti-NXP-2, anti-nuclear matrix protein-2; anti-PM/Scl, anti-polymyositis/ scleroderma; anti-SRP, anti-signal recognition particle; anti-SSA, anti-Sjögren’s syndrome A; anti-SSB, anti-Sjögren’s syndrome B; anti-TIF1-gamma, anti-transcription intermediary factor 1; CK, creatine kinase, CRP, C-reactive protein; ESR, erythrocyte sedimentation rate; EMG, electromyography; GFR, glomerular filtration rate; GGT, gamma-glutamyl transferase; IQR, interquatile range; IVIG, intravenous immunoglobulin; MAC, membrane attack complex; mRS, modified Rankin Scale; NA, not available.
